# Surgical Intervention Improves Long‐Term Survival in Stage IV Thymic Epithelial Tumors: Insights From a SEER Database Analysis

**DOI:** 10.1111/1759-7714.70030

**Published:** 2025-03-07

**Authors:** Hao Fu, Hongchao Xiong, Zhen Liang, Yunjiu Gou

**Affiliations:** ^1^ Department of Thoracic Surgery Peking University Cancer Hospital Beijing China; ^2^ Department of Thoracic Surgery Gansu Provincial Hospital Lanzhou China

**Keywords:** long‐term survival, stage IV, surgery, thymic epithelial tumors

## Abstract

**Objectives:**

This research evaluates how surgical intervention affects survival rates in individuals with stage IV thymic epithelial tumors (TET) based on data from the SEER database, offering essential information for clinical decision making.

**Methods:**

The SEER database (2004–2020) provided data on stage IV TET patients, classified into surgical and non‐surgical groups. Analytical techniques, including propensity score matching (PSM) and inverse probability treatment weighting (IPTW), were employed. The primary and secondary outcomes evaluated were cancer‐specific survival (CSS) and overall survival (OS), respectively.

**Results:**

Of 634 patients (394 diagnosed with thymoma and 240 with thymic carcinoma), 335 underwent surgery, while 299 did not. In univariate analysis, those who had surgery demonstrated significantly improved CSS and OS, with 5‐year survival rates of 74.6% for CSS and 62.3% for OS, compared to 41.4% and 26.0%, respectively, in the non‐surgical group. Multivariate analysis identified surgery as an independent factor for better CSS and OS. After applying PSM with 194 patients in each group, surgery continued to be associated with significantly improved CSS (HR = 0.417, 95% CI: 0.297–0.587, *p* < 0.001) and OS (HR = 0.457, 95% CI: 0.350–0.596, *p* < 0.001). Inverse probability of treatment weighting (IPTW) analysis confirmed these findings, showing better CSS (HR = 0.361, 95% CI: 0.265–0.492, *p* < 0.001) and OS (HR = 0.423, 95% CI: 0.335–0.535, *p* < 0.001). Subgroup analyses underscored the survival benefit of surgery for patients with stage IV thymoma and thymic carcinoma, including those with lymph node or distant metastasis.

**Conclusions:**

For stage IV thymic epithelial tumors, the inclusion of surgery in multimodal treatment can improve patient survival.

## Graphical abstract

This study utilized SEER data (2004–2020) to evaluate the impact of surgical intervention on survival in patients with stage IV thymic epithelial tumors. Among 634 patients, surgical treatment was linked to significantly better cancer‐specific survival (CSS) and overall survival (OS) compared to non‐surgical treatment, with results confirmed through propensity score matching (PSM) and inverse probability of treatment weighting (IPTW).

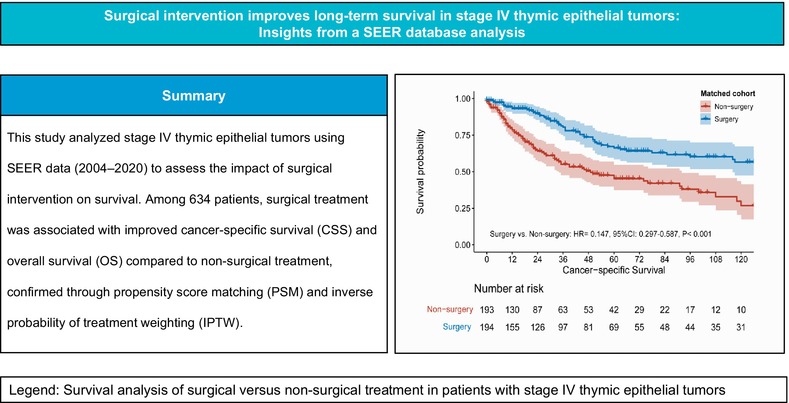

**Abbreviations**
CSS: cancer‐specific survival
IPTW: inverse probability of treatment weighting
OS: overall survival
PSM: propensity score matching
TETs: thymic epithelial tumors

## 1 Introduction

Thymic epithelial tumors (TETs), such as thymoma and thymic carcinoma, are infrequent cancers that arise from the thymus, with an incidence of about 0.15 per 100,000 people. Despite their rarity, TETs are the most prevalent tumors in the anterior mediastinum, making up approximately 40% of such tumors [1, 2]. Surgery remains the standard approach for treating early‐stage or locally advanced TETs. Both the Masaoka staging system and the 8th edition of the TNM classification categorize TETs with regional lymph node involvement and distant metastasis as stage IV [3, 4]. From an oncology standpoint, stage IV cancers generally have a poor prognosis, often necessitating systemic therapy. However, due to the relatively indolent nature of TETs, surgical intervention is frequently part of the management strategy for stage IV patients. Nonetheless, research on the effects of surgical compared to non‐surgical treatment on long‐term survival in stage IV thymoma/carcinoma is sparse, primarily because of the disease's rarity and the limited number of patients.

The SEER database offers extensive cancer statistics, encompassing diagnostic, treatment, and follow‐up information for a substantial population of cancer patients in the United States. This research uses the SEER database to evaluate the long‐term survival outcomes of surgical versus non‐surgical treatments for stage IV TETs, offering valuable insights for clinical decision‐making.

## 2 Materials and Methods

### 2.1 Ethics Statement

The SEER database is an open‐access cancer registry that provides de‐identified patient data. Consequently, utilizing the SEER database for research typically does not necessitate obtaining individual patient consent or undergoing review by an institutional review board (IRB) for ethical approval as would be required in conventional research settings.

### 2.2 Study Cohort

The data used in this study were obtained from the SEER (Surveillance, Epidemiology, and End Results) database, specifically the “Incidence‐SEER Research Data, 17 Registries, Nov 2022 Sub” dataset. The data spanning from 2004 to 2020 were selected and analyzed using SEER*Stat version 8.4.3. Inclusion criteria were: diagnosed with thymoma or thymic carcinoma; classified as stage IV according to the 8th edition of the TNM staging system. Exclusion criteria included: thymic tumors with unclear pathological diagnosis; incomplete staging and clinical information; and patients with multiple primary cancers.

In the SEER database, the 8th edition of the TNM staging for thymic epithelial malignancies began recording in 2018, with data from 2018 to 2020 directly adopted from the database. For data from 2004 to 2017, thymic epithelial tumors were restaged according to the 8th edition of the TNM staging standards based on “Stage‐Summary/Historic” and “Extent of Disease” information. “Invasive tumor confined to the site of origin,” “localized, not otherwise specified,” and “adjacent connective tissue” were defined as T1/2; “adjacent organs/structures” and “further contiguous extension” as T3/4; “regional lymph node,” “lymph nodes, not otherwise specified,” and “Regional” as N+; and “distant lymph nodes,” “distant metastasis,” “distant metastasis plus distant lymph nodes,” and “Distant” as M+ [5, 6, 7].

### 2.3 Outcomes and Definitions

Patients were categorized into a surgical and non‐surgical groups. According to the SEER data variable “RX Summ‐Surg Prim Site (1998+)”, the surgical group included: “Local tumor excision, NOS,” “Simple/partial surgical removal of primary site,” “Total surgical removal of primary site; enucleation,” “Surgery stated to be debulking,” “Radical surgery,” and “Surgery, NOS”.

The primary outcome of this study was to assess cancer‐specific survival (CSS) between the two groups, defined as the duration from tumor diagnosis to death specifically caused by thymic epithelial tumors. The secondary outcome focused on comparing overall survival (OS) between the two groups, defined as the time from tumor diagnosis to death due to any cause.

### 2.4 Statistical Analysis

Data analysis was conducted using R software (version R4.3.1). Categorical data were evaluated with either the chi‐square or Fisher's exact test, while continuous data were assessed using the t‐test. Survival outcomes were examined through Kaplan–Meier curves, with differences tested via the Log‐rank test. Multivariate analysis was performed using a Cox proportional hazards model.

Due to the retrospective design of this study, propensity score matching (PSM) was implemented with a 1:1 matching ratio to reduce differences between surgical and non‐surgical groups, accounting for variables such as treatment time, age, sex, pathological type, T stage, N stage, and M stage. Propensity scores were estimated through logistic regression using nearest neighbor matching, applying a 0.02 caliper [8]. To enhance the stability of the results, inverse probability of treatment weighting (IPTW) was employed. IPTW values were calculated by taking the inverse of the propensity score for the surgical group and the inverse of (1—PS) for the non‐surgical group [9]. The final analysis was weighted using IPTW, and a *p* value of less than 0.05 was regarded as statistically significant.

## 3 Results

### 3.1 Baseline Characteristics

**FIGURE 1:**
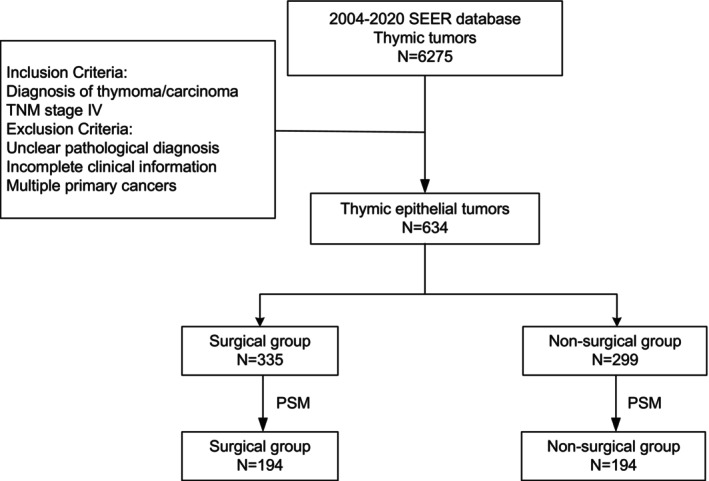
Patient enrollment flowchart. PSM: propensity score matching.

This study included 634 patients who met the inclusion and exclusion criteria (Figure 1). The participants had a median age of 59 years, with an interquartile range of 49 to 69 years. Among them, 367 were male and 267 were female. Of these, 394 had thymomas and 240 had thymic carcinoma. The surgical group included 335 patients, while 299 patients were in the non‐surgical group. Over the period from 2004 to 2020, the proportion of surgical to non‐surgical patients remained relatively stable (Table 1).

**TABLE 1:** Fundamental details before and after matching propensity scores for the surgical and non‐surgical groups.

Items	Levels	Before PSM			After PSM		
		Non‐surgery	Surgery	*p*	Non‐surgery	Surgery	*p*
		299	335		194	194	
Sex, *n* (%)	Female	126 (42.1)	141 (42.1)	1	82 (42.3)	69 (35.6)	0.211
	Male	173 (57.9)	194 (57.9)		112 (57.7)	125 (64.4)	
Age, *n* (%)	≤ 59 years	131 (43.8)	188 (56.1)	0.003	85 (43.8)	91 (46.9)	0.61
	> 59 years	168 (56.2)	147 (43.9)		109 (56.2)	103 (53.1)	
Race, *n* (%)	Asia	57 (19.1)	70 (20.9)	0.271	34 (17.5)	33 (17.0)	0.978
	Black	63 (21.1)	54 (16.1)		35 (18.0)	34 (17.5)	
	White	179 (59.9)	211 (63.0)		125 (64.4)	127 (65.5)	
Pathology, *n* (%)	Thymic carcinoma	139 (46.5)	101 (30.1)	< 0.001	69 (35.6)	75 (38.7)	0.599
	Thymoma	160 (53.5)	234 (69.9)		125 (64.4)	119 (61.3)	
T stage, *n* (%)	T1/2	109 (36.5)	77 (23.0)	< 0.001	53 (27.3)	55 (28.4)	0.91
	T3/4	190 (63.5)	258 (77.0)		141 (72.7)	139 (71.6)	
N stage, *n* (%)	N+	159 (53.2)	145 (43.3)	0.016	97 (50.0)	97 (50.0)	1
	N0	140 (46.8)	190 (56.7)		97 (50.0)	97 (50.0)	
M stage, *n* (%)	M+	230 (76.9)	237 (70.7)	0.094	135 (69.6)	135 (69.6)	1
	M0	69 (23.1)	98 (29.3)		59 (30.4)	59 (30.4)	
Treatment time, *n* (%)	2004–2009	85 (28.4)	122 (36.4)	0.095	64 (33.0)	73 (37.6)	0.167
	2010–2014	105 (35.1)	108 (32.2)		66 (34.0)	49 (25.3)	
	2015–2020	109 (36.5)	105 (31.3)		64 (33.0)	72 (37.1)	

Abbreviation: PSM: propensity score matching.

### 3.2 Survival Analysis

**FIGURE 2:**
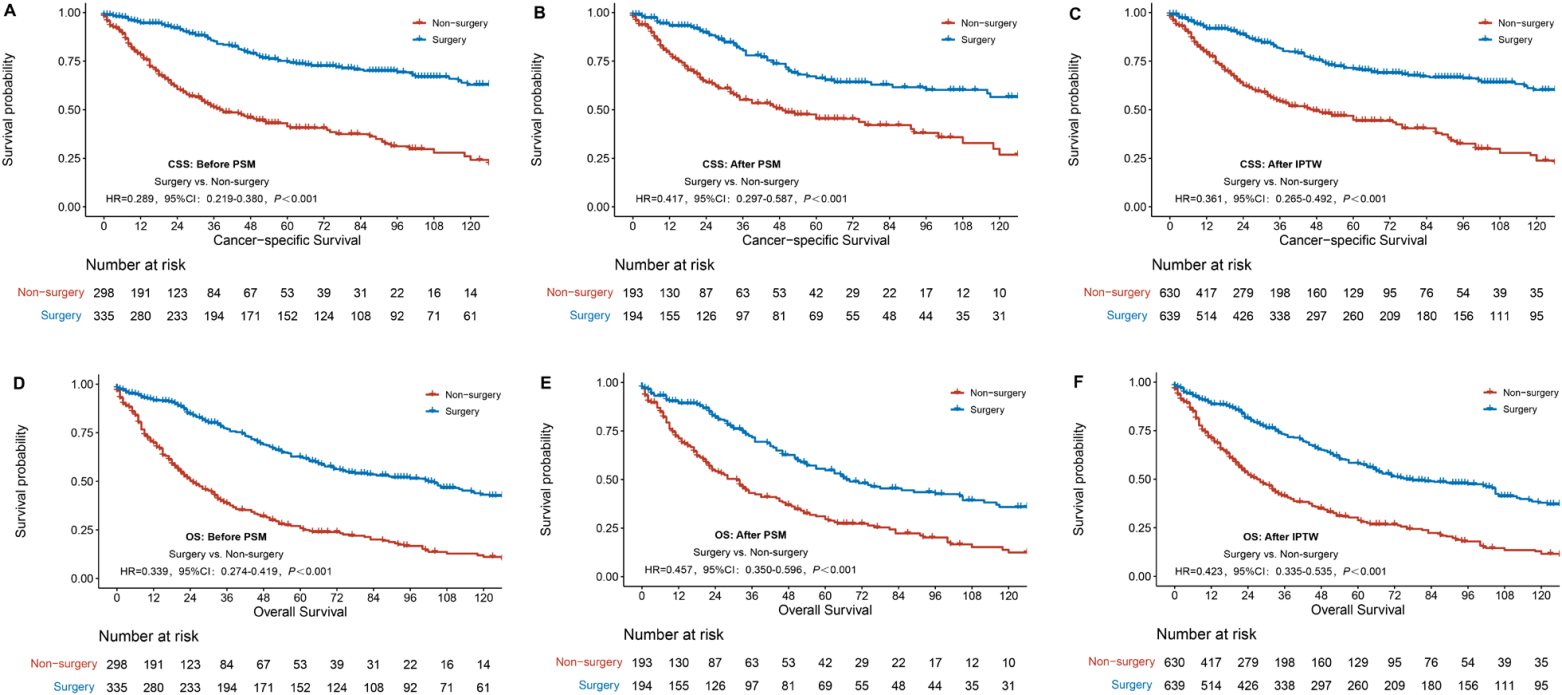
Survival comparison curves between the surgical and non‐surgical groups under different statistical models. PSM: propensity score matching; IPTW: inverse probability of treatment weighting.

The cancer‐specific survival (CSS) rates for the cohort were 86.9% at 1 year, 69.9% at 3 years, and 59.9% at 5 years. The overall survival (OS) rates were 81.4%, 58.9%, and 45.4% at 1, 3, and 5 years, respectively. Patients in the surgical group exhibited superior 5‐year CSS and OS than the non‐surgical group, with a CSS of 74.6% versus 41.4% (HR = 0.289, 95% CI 0.219–0.380, *p* < 0.001) and an OS of 62.3% versus 26.0% (HR = 0.399, 95% CI 0.274–0.419, *p* < 0.001) (Figure 2).

The multivariate analysis identified surgical intervention (HR = 0.331, 95% CI 0.247–0.442, *p* < 0.001) and pathological type (HR = 0.414, 95% CI 0.312–0.548, *p* < 0.001) as independent prognostic indicators for CSS in stage IV thymic malignancies, accounting for variables such as treatment time, age, sex, race, T stage, N stage, and M stage (Table 2).

**TABLE 2:** Cancer‐specific survival analysis between the surgical and non‐surgical groups before and after PSM and after IPTW.

Items	Analysis	HR (surgery vs. non‐surgery)	95% CI	*p*
Before PSM	Univariate	0.289	0.219–0.380	< 0.001
Before PSM	Multivariable	0.331	0.247–0.442	< 0.001
After PSM	Univariate	0.417	0.297–0.587	< 0.001
After PSM	Multivariable	0.408	0.290–0.576	< 0.001
IPTW	Univariate	0.361	0.264–0.492	< 0.001
IPTW	Multivariable	0.343	0.252–0.467	< 0.001

Abbreviations: CI: confidence interval; HR: hazard ratio; IPTW: inverse probability of treatment weighting; PSM: propensity score matching.

### 3.3 Propensity Score Matching (PSM)

Because the baseline characteristics of the two groups were not balanced, a 1:1 propensity score matching was performed based on factors including treatment time, age, sex, race, pathological type, T stage, N stage, and M stage. After matching, Both groups, surgical and non‐surgical, had 194 patients, and their clinical information was balanced (Table 1). Univariate analysis revealed that the surgical group had superior 5‐year cancer‐specific survival and overall survival compared to the non‐surgical group, with CSS rates of 66.2% versus 45.3%, and OS rates of 54.8% versus 29.6% (CSS: HR = 0.417, 95% CI 0.297–0.587, *p* < 0.001; OS: HR = 0.457, 95% CI 0.350–0.596, *p* < 0.001) (Figure 2).

Multivariate analysis, performed post‐PSM, revealed that surgical intervention (HR = 0.408, 95% CI 0.290–0.576, *p* < 0.001) and the type of pathology independently influenced CSS outcomes in stage IV thymic malignancies (Table 2).

### 3.4 Inverse Probability of Treatment Weighting (IPTW)

Inverse probability weighting was used to balance the weights of baseline characteristics, including treatment time, race, age, sex, pathological type, T stage, N stage, and M stage. Univariate analysis showed that patients in the surgical group had superior CSS compared to those in the non‐surgical group, with an HR of 0.361 (95% CI: 0.265–0.492, *p* < 0.001); Additionally, patients in the surgical group exhibited better OS compared to the non‐surgical group, with an HR of 0.423 (95% CI: 0.335–0.535, *p* < 0.001) (Figure 2). Multivariate analysis revealed that, for CSS, surgical intervention, pathological type, and presence of distant metastasis were independent prognostic indicators (Table 2). For OS, surgical intervention, age, pathological type, and distant metastasis were identified as significant prognostic factors (Table 3).

**TABLE 3:** Overall survival analysis between the surgical and non‐surgical groups before and after PSM and after IPTW.

Items	Analysis	HR (surgery vs. non‐surgery)	95% CI	*p*
Before PSM	Univariate	0.339	0.274–0.419	< 0.001
Before PSM	Multivariable	0.398	0.318–0.499	< 0.001
After PSM	Univariate	0.457	0.350–0.596	< 0.001
After PSM	Multivariable	0.411	0.316–0.536	< 0.001
IPTW	Univariate	0.423	0.335–0.535	< 0.001
IPTW	Multivariable	0.402	0.320–0.504	< 0.001

Abbreviations: PSM: propensity score matching; IPTW: inverse probability of treatment weighting; HR: hazard ratio; CI: confidence interval.

### 3.5 Subgroup Analysis

**FIGURE 3:**
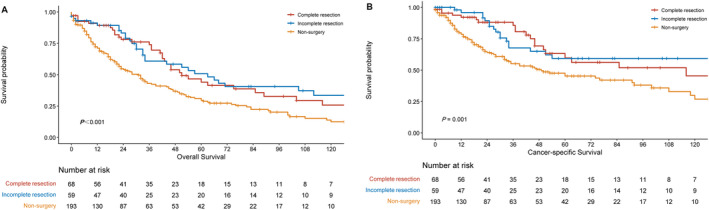
Comparison of cancer‐specific survival (CSS) (A) and overall survival (OS) (B) among patients with complete resection, incomplete resection, and non‐surgical treatment.

In the matched data for the surgical group, 68 cases had complete resection, 59 had incomplete resection, and 67 had an uncertain resection status. According to survival analysis, even cases with incomplete resection had better overall survival and cancer‐specific survival outcomes than non‐surgical patients (Figure 3). The 5‐year OS rates for complete resection, incomplete resection, and non‐surgery are 59.7%, 59.3%, and 45.4%, respectively, with a *p* value of 0.001; The 5‐year OS rates are 44.1%, 50.7%, and 29.5%, respectively, with a *p* < 0.001.

**FIGURE 4:**
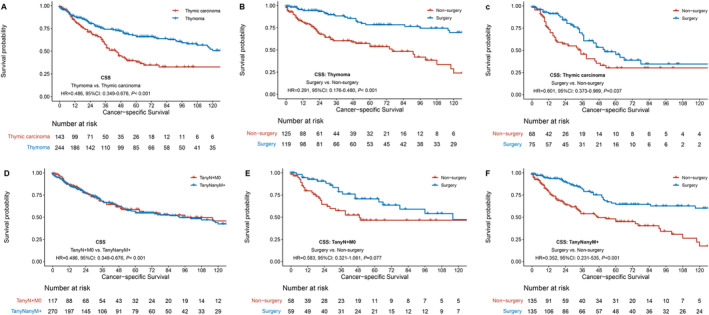
Survival analysis of surgical versus non‐surgical groups in stage and pathological subgroups.

The matched data were divided into thymoma and thymic carcinoma subgroups according to pathological type. For stage IV thymoma patients, the surgical group demonstrated superior CSS and OS compared to the non‐surgical group, with 5‐year CSS rates of 78.4% versus 53.8%, HR = 0.291, 95% CI: 0.176–0.480, *p* < 0.001; The 5‐year OS rates of 68.9% versus 33.4%, HR = 0.344, 95% CI: 0.239–0.496, *p* < 0.001 (Figure 4). Similarly, for patients with stage IV thymic carcinoma, surgery also conferred a survival benefit, with 5‐year CSS rates of 45.3% versus 30.1%, HR = 0.601, 95% CI: 0.373–0.969, *p* = 0.037; The 5‐year OS rates of 32.1% versus 22.3%, HR = 0.691, 95% CI: 0.461–1.033, *p* = 0.068 (Figure 4).

Based on metastatic sites, the matched patients were categorized into lymph node metastasis (T_any_N + M0) and distant metastasis (T_any_N_any_M+) groups, with no significant differences observed in CSS and OS between the two groups (CSS: HR = 1.054, 95% CI: 0.740–1.503, *p* = 0.770; OS: HR = 1.184, 95% CI: 0.887–1.580, *p* = 0.252) (Figure 4). For stage IV thymic epithelial tumors with lymph node metastasis, CSS in the surgical group was superior compared to the non‐surgical group, but not statistically significant, with 5‐year CSS rates of 70.6% versus 46.6%, HR = 0.583, 95% CI: 0.321–1.061, *p* = 0.077. The surgical group also had better OS, with 5‐year OS rates of 59.2% versus 30.0%, HR = 0.544, 95% CI: 0.331–0.895, *p* = 0.017. For those with distant metastasis, the surgical group shows superior CSS and OS than the non‐surgical group, with 5‐year CSS rates of 64.5% versus 45.1%, HR = 0.352, 95% CI: 0.231–0.535, *p* < 0.001; The 5‐year OS rates of 53.1% versus 29.3%, HR = 0.414, 95% CI: 0.301–0.570, *p* < 0.001 (Figure 4).

## 4 Discussion

Surgical resection is the standard treatment for early or locally advanced thymoma/carcinoma, providing reliable long‐term outcomes. Unlike most solid malignancies, thymic tumors exhibit relatively indolent biological behavior, and surgery is often included in the treatment plan for stage IV disease. However, in these cases, surgery is typically palliative, and the benefit of surgery for stage IV patients has long been debated, with no supporting data to date. Previous studies with small sample sizes have primarily focused on stage IV patients with pleural or pericardial metastases [10, 11, 12]. Kaba et al. [13] reported on the efficacy of surgery in 39 patients with Masaoka stage IV thymoma, with a median survival of 132 months and 3‐year, 5‐year, and 10‐year survival rates of 93%, 93%, and 56%, respectively. Okuda et al. [14] analyzed data from the Japanese National Cancer Database, showing that among 118 patients with stage IVa thymoma, Those who experienced a complete resection had higher survival rates than those with an incomplete resection, with 5‐year and 10‐year survival rates of 88.6% and 84.5%, compared to 88.6% and 46.3%, respectively (*p* = 0.037), supporting the efficacy of surgery for such patients. A meta‐analysis on debulking surgery for thymoma was carried out by Hamaji et al. [15], including 13 studies, showing superior survival in 172 patients who had debulking surgery compared to 142 who only underwent surgical biopsy. These studies were small in scale and lacked prospective research. In this study, 630 stage IV thymic epithelial tumor patients from the SEER database were examined, and it was found that surgery led to better survival outcomes. For the surgical group, the 5‐year cancer‐specific and overall survival rates were 74.6% and 41.4%, compared to 62.3%and 26.0% for the non‐surgical group.

This study analyzed the changes in the proportion of surgical patients over different periods. From 2004 to 2020, the proportion of patients undergoing surgery remained relatively stable across time intervals (36.4% in 2004–2009, 32.2% in 2010–2014, and 31.3% in 2015–2020). This stability suggests that surgical indications for stage IV thymic epithelial malignancies have not significantly expanded over the past decade. Therefore, surgical decisions are likely based more on the individual patient's condition and physician evaluation than on changes in medical policies or guidelines. This finding indicates that surgical selection remains stringent, possibly focused on patients with a higher likelihood of benefit. Although the proportion of surgeries has not notably increased, advancements in surgical techniques and perioperative management may have occurred over the decade. For example, more advanced minimally invasive surgeries and more refined multidisciplinary treatment (MDT) approaches may have improved survival rates for patients undergoing surgery in later years. Consequently, treatment time intervals were included as a variable in this study.

To reduce bias between the two groups, this study used both propensity score matching (PSM) and inverse probability of treatment weighting (IPTW) to validate factors potentially affecting survival. Matching factors included treatment time, age, race, sex, pathological type, N stage, and M stage.

After matching, both groups consisted of 194 patients, with balanced clinical information, and the results still revealed that the surgical group had superior survival than the non‐surgical group. Multivariate analysis, both before and after matching, confirmed that patients in the surgical group had superior survival, identifying surgery as a favorable independent prognostic factor for stage IV thymic epithelial tumors. Given that thymic carcinoma has a significantly poorer prognosis than thymoma, a subgroup analysis was conducted based on pathological type. The analysis demonstrated that, for both stage IV thymic carcinoma and thymoma, the surgical group had superior survival than the non‐surgical group. The IPTW analysis yielded conclusions consistent with the PSM findings.

Both the 8th edition of the AJCC/UICC TNM staging system and the traditional Masaoka staging system classify lymph node metastasis and distant metastasis as stage IV. However, the rationale for this classification has long been questioned, as many scholars assume that stage IV patients with lymph node metastasis have superior survival compared to those with distant organ metastasis. Yang et al. [16] reported on 68 cases of stage IV thymic carcinoma, showing that patients with lymph node metastasis had superior survival compared to those with distant metastasis, with median survival times of 40 months and 20 months, respectively. In our study, patients were divided into subgroups of lymph node metastasis and distant metastasis. No significant difference was found between the two groups in the survival analysis, with 5‐year overall survival rates of 41.4% and 47.5%, respectively. Subgroup analysis by metastatic site indicated that, regardless of lymph node or distant metastasis, patients in the surgical group had better CSS and OS than those in the non‐surgical group.

### 4.1 Limitations

Although this study is the first large‐sample analysis to compare the effect of surgery on long‐term survival in stage IV thymoma/carcinoma, using PSM and IPTW to control for biases related to key clinical factors, some limitations remain: (1) This is a retrospective study, which inherently limits the ability to establish causality; (2) The SEER database lacks detailed surgical information, including data on the specific sites of metastasis; (3) Comprehensive data on the locations and numbers of distant metastases are not available, hindering a more precise understanding of tumor spread and its impact on survival outcomes; (4) While our study demonstrates that surgery significantly improves cancer‐specific survival (CSS) and overall survival (OS) in stage IV thymic epithelial malignancy patients, the SEER database does not include preoperative performance status (PS) data. This lack of PS information introduces potential selection bias, as patients with better preoperative physical status are more likely to undergo surgery, while those in poorer condition may be directed toward non‐surgical or palliative treatments. Future studies should incorporate preoperative PS data to more comprehensively assess patient conditions and reduce selection bias.

## 5 Conclusion

In conclusion, our results suggest that surgery, as part of a multimodal approach, improves both cancer‐specific survival (CSS) and overall survival (OS) in patients with stage IV thymic epithelial tumors. Surgery may be considered as part of the multimodal treatment for selected patients.

## Author Contributions

**Hao Fu:** data curation; formal analysis; writing – original draft; writing – review and editing. **Hongchao Xiong:** investigation; methodology; supervision; writing – review and editing. **Zhen Liang:** conceptualization; investigation; supervision; writing – review and editing. **Yunjiu Gou:** conceptualization; investigation; methodology; supervision; writing – review and editing.

## Conflicts of Interest

The authors declare no conflicts of interest.

## Data Availability

The data underlying this article will be shared by the corresponding author on reasonable request.
